# Comparison of Prothrombin Time Derived From CoaguChek XS and Laboratory Test According to Fibrinogen Level

**DOI:** 10.1002/jcla.21722

**Published:** 2014-03-28

**Authors:** Sue Jung Kim, Eun Young Lee, Rojin Park, Juwon Kim, Jaewoo Song

**Affiliations:** ^1^ Department of Laboratory Medicine Yonsei University College of Medicine Seoul Korea; ^2^ Department of Laboratory Medicine Soon Chun Hyang University Hospital Seoul Korea; ^3^ Department of Laboratory Medicine Yonsei University Wonju College of Medicine Wonju Korea

**Keywords:** prothrombin time, INR, fibrinogen, point‐of‐care, CoaguChek XS

## Abstract

**Background:**

CoaguChek XS is one of the most widely used point‐of‐care (POC) devices to evaluate prothrombin time for monitoring oral anticoagulant therapy. Unlike laboratory methods, it detects electrical signals produced by thrombin activity to derive the international normalized ratio (INR). Therefore, we hypothesized that laboratory methods and CoaguChek XS could produce different results according to fibrinogen level.

**Methods:**

We compared INR values obtained from the CoaguChek XS and conventional laboratory method with 91 plasma samples covering a wide range of fibrinogen levels.

**Results:**

The samples were stratified into low, mid, and high fibrinogen groups by fibrinogen levels of <130 mg/dl, 130–450 mg/dl, and >450 mg/dl, respectively. The mean INR difference of the low fibrinogen group was significantly different from that of the mid or high fibrinogen group (*P* < 0.001). In the low fibrinogen group, CoaguChek XS INR showed a negative bias compared with the laboratory INR, while the mid and high fibrinogen groups had positive bias.

**Conclusion:**

Our results suggest that patient selection according to fibrinogen status should precede the implementation of POC testing using CoaguChek XS. Also, periodic comparisons between CoaguChek XS and laboratory INR results should be continued during the use of CoaguChek XS.

Prothrombin time/international normalized ratio (PT/INR) is used for dosing and monitoring oral anticoagulant therapy (OAT) based on warfarin 1. The therapeutic range of warfarin is narrow and there is a considerable variation in inter‐ and intraindividual pharmacologic response. Therefore, proper monitoring of the dose response is essential for prevention of unwanted bleeding or recurring thrombosis 1. Implementation of point‐of‐care (POC) devices to measure INR for patient self‐testing or even patient self‐management is now suggested as a useful solution to the increasing need for OAT care facilities. With this background, there have been a large number of publications evaluating the performance of many kinds of POC INR devices 2, 3, 4, 5, 6, 7, 8. The accordance between POC INR and clinical laboratory‐based INR values has been repeatedly reported to be satisfactory, and monitoring based on patient self‐testing is now generally accepted as a reliable way to continue OAT 9. However, in several previous studies, individual cases showing unusual differences between POC and laboratory INR have not been examined in enough detail to be clearly explained 4, 5, 8, 10.

Various methods have been adopted to derive the INR value for POC devices, and electrochemical detection of thrombin activity is one of the most widely used assay principles. The CoaguChek XS (Roche Diagnostics, Mannheim, Germany) measures the amount or kinetics of thrombin activity by detecting the electrical signal produced when the peptide substrate Electrocyme TH is cleaved by thrombin 11. Most laboratory INRs are derived from PT that relies on clot formation, which in turn can be influenced by fibrinogen, the main structural component of clot. Therefore, we hypothesized that the discrepancy between CoaguChek XS and laboratory INRs could appear in different patterns depending on the fibrinogen level of the applied specimen. It is possible that the added bleeding risk from an abnormality of fibrinogen cannot be detected by INR using the same kind of POC. Few data have been published on the performance of CoaguChek XS INR in patients with low fibrinogen level. In this study, we compared INR values obtained from CoaguChek XS and conventional laboratory method based on clotting time from a group of plasma samples covering a wide range of fibrinogen levels.

Ninety‐one sodium citrate (3.2%) venous blood samples submitted for fibrinogen testing to the clinical laboratory at Severance Hospital in Seoul, Korea, were collected. Platelet‐poor plasma was obtained by centrifugation for 15 min at 2,000 × *g*. The fibrinogen level was measured using Fibrinogen‐C XL reagent (Instrumentation Laboratory, Milan, Italy) via Clauss method using ACL TOP (Beckman Coulter, Fullerton, CA). Samples were selected after fibrinogen testing according to the fibrinogen level. PT INR was measured by both the laboratory method using ACL TOP with RecombiPlasTin 2G reagent (Instrumentation Laboratory) and CoaguChek XS. Because CoaguChek XS requires samples without calcium depletion (originally capillary blood), citrate plasma was recalcified with calcium chloride (1:1) for the PT INR test by CoaguChek XS. The INR difference was calculated by subtracting the laboratory INR from the CoaguChek XS INR. The %INR difference used the following formula: 100(CoaguChek XS INR − laboratory INR)/laboratory INR.

Statistical analysis was performed using IBM SPSS Statistics version 20 (SPSS Inc., Chicago, IL). Pearson's correlations were performed to compare the CoaguChek XS and the laboratory INR. ANOVA test with Bonferroni post‐test and Welch's *F*‐test with post hoc comparisons were performed to compare INR differences among different fibrinogen groups. A *P* value less than 0.05 was regarded as significant. This study was approved by the institutional review board of Severance Hospital.

The laboratory INR results ranged from 0.71 to 7.44 with a mean of 1.65, and CoaguChek XS INR ranged from 1.0 to 7.7 with a mean of 1.8. The overall paired values from the CoaguChek XS and laboratory method showed good correlation (*r* = 0.97, *P* < 0.001, Fig. 1A). Sixty‐three of 91 pairs (69.2%) showed a difference within ±0.2, and 82 of 91 (90.1%) were within ±0.4, both of which have been considered as acceptable limits for accuracy in previous evaluation studies. The mean INR difference was 0.12, and the range was 0.7–1.7 (Fig. 1B and C). The mean %INR difference was 11% and ranged from −19% to 55% (Fig. 1D). The INR differences as a function of the mean INR did not show significant correlation (*r* = 0.09, *P* = 0.41, Fig. 1B). The INR differences between the two methods showed significant correlation with fibrinogen level (*r* = 0.47, *P* < 0.001, Fig. 1C). The %INR differences also were significantly correlated with fibrinogen level (*r* = 0.28, *P* = 0.007, Fig. 1D). Samples were divided into three groups according to the fibrinogen level and also considering the graphical distribution on INR difference versus fibrinogen plot and reference range of fibrinogen. These three groups were designated as the low, mid, and high groups with fibrinogen levels <130 mg/dl, 130–450 mg/dl, and >450 mg/dl, respectively. Twenty‐three patients in the low fibrinogen group with fibrinogen levels 34–129 mg/dl showed a −0.14 mean INR difference (range: −0.70 to 0.17). The mid fibrinogen group consisted of 34 patients showed fibrinogen level from 135 to 446 mg/dl and had a 0.23 mean INR difference (range: 0.05–0.76). The mean INR difference was 0.20 (range: 0.07–1.70) in the high fibrinogen group of 34 patients with fibrinogen levels from 453 to 1428 mg/dl. The low fibrinogen group was significantly different in mean INR difference and mean %INR difference compared to the mid group (*P* < 0.001) and the high group (*P* < 0.001). There was no significant difference in the mean INR difference and mean %INR differences between the mid and high fibrinogen groups (*P* = 1.00 and *P* = 0.53). The low fibrinogen group deviated to a negative value, while positive biases were observed in the mid and high group (Fig. 1C and D). We looked for a discrepancy in clinical decisions, assuming that the target INR was 2.0–3.0 as an example, which is commonly adopted as the therapeutic range of warfarin 1. There were four discrepant cases that would have caused false clinical decision (Fig. 1A). Three of them had low fibrinogen levels, and the laboratory method produced higher INR value than the CoaguChek XS.

**Figure 1:**
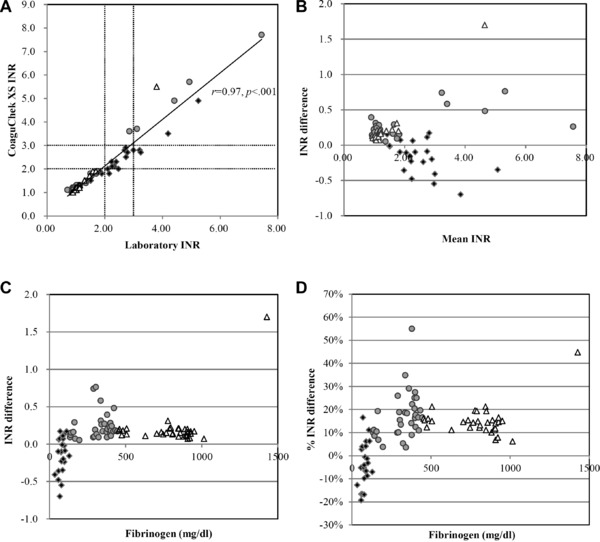
CoaguChek XS INR and laboratory INR results were plotted according to the fibrinogen level: low fibrinogen group (rhombus), mid fibrinogen group (circle), and high fibrinogen group (triangle). (A) Correlation between CoaguChek XS INR and laboratory INR (*r* = 0.97, *P* < 0.001). An arbitrary therapeutic range of warfarin is indicated by a dotted line. (B) Differences between the CoaguChek XS INR and the laboratory INR according to the mean INR value. The absolute differences showed a positive correlation with mean INR value (*r* = 0.58, *P* < 0.001). The INR difference was calculated as by subtracting the laboratory INR from the CoaguChek XS INR, and the mean INR was determined by adding the CoaguChek XS INR to the laboratory INR and then dividing the sum by 2. (C) Correlation between INR differences and fibrinogen level (*r* = 0.47, *P* < 0.001). (D) Correlation between %INR differences and fibrinogen level (*r* = 0.28, *P* = 0.007). Percent differences between CoaguChek XS INR and laboratory INR according to the mean INR value. The %INR difference was calculated with the following formula: 100(CoaguChek XS INR − laboratory INR)/laboratory INR. INR, international normalized ratio.

Although the overall INR obtained by POC and laboratory methods showed a good correlation, CoaguChek XS INR showed bidirectional biases from laboratory INR values depending on the fibrinogen level. Our results indicate that the use of CoaguChek XS INR without meticulous matching to laboratory INR may cause problems in certain conditions associated with hypofibrinogenemia. Hypofibrinogenemia can be associated with various medical conditions such as hemodilution, blood loss, disseminated intravascular coagulation, sepsis, and chronic liver diseases 12. Chronic conditions such as liver disease can certainly accompany thrombophilic conditions and may require OAT. Also, POC INR devices can be used for hospital inpatients with the intention of reducing the volume of blood sampling or shortening the turnaround time of screening coagulation tests 13, 14, 15. Such cases can often be complicated by acute and severe medical conditions associated with fibrinogen abnormalities. Therefore, in such conditions, depending solely on the CoaguChek XS INR devices for clinical decision should only be considered with much caution.

Two potential problems should be pointed out in our study in regard to the samples used in comparison. First, citrate plasma was used both for the CoaguChek XS and laboratory INRs despite the fact that the CoaguChek XS is designed to use capillary blood from a finger prick. However, it has been repeatedly reported that plasma has been used successfully for external quality assessment purposes to detect discrepancies between these devices 16, 17, 18, 19. Also, lyophilized plasma has been shown to be a desirable option for use as a material for internal quality control of several kinds of POC INR devices 20. Second, in order to evaluate the samples with a wide range of fibrinogen levels, the participants of this study were not confined to patients on OAT. Although POC INR is primarily intended for use in patients requiring OAT, the use of POC INR is not limited to OAT monitoring. POC‐INR devices are reportedly used as a substitute for routine coagulation screening tests, especially in the pediatric field where it is often difficult to obtain sufficient venous blood samples 15. In the surgical and critical care fields, the same types of POC devices have been shown to be equivalent to laboratory PT when deciding if an transfusion is needed; they were introduced as a useful tool to rapidly detect coagulopathies on site or intraoperatively 13, 14. With the expanding use of POC INR devices in medical fields other than OAT, we decided that beyond‐OAT citrate plasma with varying fibrinogen levels could reasonably serve our purposes.

In conclusion, our results suggest that patient selection according to fibrinogen status should precede the implementation of POC testing using CoaguChek XS. Periodic comparison between CoaguChek XS and laboratory INR should be continued during the use of CoaguChek XS. Care also should be taken to interpret the CoaguChek XS INR results with purposes other than OAT monitoring.

## CONFLICT OF INTEREST

The authors report nonfinancial support from Roche Diagnostics Korea during the conduct of the study. Roche Diagnostics Korea has supported CoaguChek XS strips used in this study.
